# Family Pet Ownership during Childhood: Findings from a UK Birth Cohort and Implications for Public Health Research

**DOI:** 10.3390/ijerph7103704

**Published:** 2010-10-18

**Authors:** Carri Westgarth, Jon Heron, Andy R. Ness, Peter Bundred, Rosalind M. Gaskell, Karen P. Coyne, Alexander J. German, Sandra McCune, Susan Dawson

**Affiliations:** 1 School of Veterinary Science, Faculty of Health and Life Sciences, University of Liverpool, Leahurst Campus, Neston, Cheshire, CH64 7TE, UK; E-Mails: rosgask@liverpool.ac.uk (R.M.G.); kpcoyne@liverpool.ac.uk (K.P.C.); ajgerman@liverpool.ac.uk (A.J.G.); dawson@liv.ac.uk (S.D.); 2 Department of Social Medicine, University of Bristol, 39 Whatley Road, Bristol, BS8 2PS, UK; E-Mail: Jon.Heron@bristol.ac.uk (J.H.); 3 School of Oral and Dental Science, University of Bristol, Lower Maudlin Street, Bristol, BS1 2LY, UK; E-Mail: Andy.Ness@bristol.ac.uk (A.R.N.); 4 School of Population, Community and Behavioural Sciences, University of Liverpool, Brownlow Hill, Liverpool, L69 3GB, UK; E-Mail: peterb@liverpool.ac.uk (P.B.); 5 WALTHAM^®^ Centre for Pet Nutrition, Waltham-on-the-Wolds, Melton Mowbray, Leics, LE14 4RT, UK; E-Mail: sandra.x.mccune@eu.effem.com (S.M.)

**Keywords:** ALSPAC, pet, dog, cat, child

## Abstract

In developed nations, approximately half of household environments contain pets. Studies of Human-Animal Interaction (HAI) have proposed that there are health benefits and risks associated with pet ownership. However, accurately demonstrating and understanding these relationships first requires a better knowledge of factors associated with ownership of different pet types. A UK birth cohort, the Avon Longitudinal Study of Parents and Children (ALSPAC), were used to collect pet ownership data from the mothers, from gestation to child age 10 years old. 14,663 children were included in the study, of which mothers of 13,557 reported pet information at gestation, and 7,800 by age 10. Pet types recorded include cat, dog, rabbit, rodent, bird, fish and tortoise/turtle. The dataset also contains a number of demographic, socioeconomic and behavioural variables relevant to human health behaviour. Logistic regression was used to build multivariable models for ownership of each pet type at age 7 years. Family pet ownership increased during childhood, in particular rabbits, rodents and fish. A number of socioeconomic and demographic factors were associated with ownership of different pet types and the effects differed depending on the pet type studied. Variables which require consideration by researchers include gender, presence of older siblings, ethnicity, maternal and paternal education, maternal and paternal social class, maternal age, number of people in the household, house type, and concurrent ownership of other pets. Whether the mother had pets during her childhood was a strong predictor of pet ownership in all models. In HAI studies, care should be taken to control for confounding factors, and to treat each pet type individually. ALSPAC and other similar birth cohorts can be considered a potential resource for research into the effects of pet ownership during childhood.

## 1. Introduction

The demographics of pet ownership are of interest to those working in the fields of public health, psychology, veterinary and social sciences. The study of companion animal ownership on the physical, social and psychological health of people is an expanding field of research, encompassed by the term ‘Human-Animal Interactions’ (HAI). Considering that around half of people in developed nations own pets, the area has received little scientific attention [1].

There is some evidence to suggest that pets confer both physiological and psychological health benefits [2–6]. Pet ownership may provide emotional protection from the stresses and strains of life (the ‘buffer’ effect) [3] and/or it may encourage a more active lifestyle [7]. Compared to those who do not own pets, pet owners report fewer visits to the doctors [8] and have improved survival following heart attack [9]. Katcher and Friedmann (1982) suggested seven important functions of pet ownership: companionship; something to care for; something to touch and fondle; something to keep one busy; a focus of attention; exercise and safety. Contact with companion animals has been suggested to have important roles in enhancing child development [5,10,11], in the wellbeing of older people [5,11,12] and may also be used in a therapeutic setting [10,12,13].

Of particular recent interest to public health researchers has been the relationship between dog ownership and physical activity. It has been hypothesised that ownership of a dog results in increased physical activity due to dog walking. A number of studies have suggested that increased physical activity in adults does occur through walking your dog [7,14–21] Furthermore, one study has suggested that young children (5–6 yrs) were less likely to be obese if they owned a dog [22].

There may also be risks associated with pet ownership, including behavioural problems, aggression and bites, relinquishment of unwanted pets, allergies and zoonosis [1,3]. These not only affect immediate household members, but also the wider community [1]. Some groups in human society are at greater risk of zoonotic infection due to their immune system or behaviour; for example young children, the elderly, pregnant women and the immuno-compromised [23,24].

The major challenges to HAI research are the methodological difficulties encountered in studying this complex relationship and assigning direction of causation [2,25]. Demographic variables such as age, gender, socioeconomics and ethnic status are known to be associated with many health behaviours [26]. Taking the study of health benefits of dog walking as an example, there may be differences between people who own dogs and people who do not, leading to apparent associations with health outcomes that are not truly causal, for example, healthier, more active people may be more likely to acquire a dog. Few HAI studies have been able to control adequately for possible confounding factors during design or analysis [7,27]. The methodology is often flawed and sample sizes too small to support the conclusions drawn [28]. With increasing evidence for socioeconomic and demographic differences in pet owners versus non-owners, [29–33], this is of concern.

If we are to examine the evidence for health benefits (and risks) of pet ownership, we first need to understand the factors associated with pet ownership. We need to better describe which socioeconomic, demographic and behavioural variables are associated with ownership of different pet types, so that they can be controlled for as much as possible during study design and analysis of data involving HAI. Previous studies have tended to study ‘pets’ in general, or at most differentiated dogs from cats. When dogs and cats are considered separately, differences in the type of people who own them are seen [29–31,33], including; social class, education level, household composition, gender of respondents, and house type. It is likely that there are also differences between factors associated with ownership of other pet types.

Many human-animal interaction studies are cross-sectional in nature, thus lacking the longitudinal data required to interpret directionality of the causation [2,25]; therefore longitudinal studies that include pet ownership, for example birth cohorts, could be of great value to the field. Pet ownership data may be collected in longitudinal studies of health, often for the purpose of research into asthma and allergies [34]. Many existing birth cohorts are large, negating the problem of small sample sizes. In addition, they collect pet data prospectively, rather than assuming reliable recall a number of years later; shown to be reasonable, but not perfect [35]. For these reasons, birth cohorts offer unique opportunities for HAI research, but have been underutilised thus far.

Epidemiological studies have suggested that pets are more common in families with children than those without children [29–31,33,36–46]. Despite this, there are no published longitudinal studies describing pet ownership during childhood; children may be particularly interesting to study as they can have close relationships with their pets, but ownership is controlled by their parents.

This study uses a large UK birth cohort (the Avon Longitudinal Study of Parents and Children—ALSPAC), to describe pet ownership during childhood. It recruited women in pregnancy and followed them and their children. Pet ownership was reported up to age 10 years and was reported by the mother (or primary carer) of the child, rather than the child itself, as prospective data on infants requires report by others [35].

The aims of this study were to:

1. Describe prevalence of family ownership of different pet types, and how these change, up to age 10 years.
2. Describe the factors that appear to be associated with each pet type, up to age 10 years.
3. Derive a multivariable model for ownership of each pet type at age 7 years.

This will identify possible confounding variables to be accounted for during future HAI research using this dataset and others. Age seven was chosen because from this age all children were invited to attend clinics at which anthropometric data were collected, such as their height and weight.

## 2. Experimental Section

### 2.1. Data Collection

ALSPAC is a prospective study that has been described in detail elsewhere [47]. ALSPAC recruited 14,541 pregnant women resident in Avon, UK with expected dates of delivery 1st April 1991 to 31st December 1992. After exclusion of those without a known live birth outcome, and exclusion of triplets and quadruplets, the full dataset used in this paper was for 14,663 fetuses.

ALSPAC has collected data from pregnancy onwards using postal questionnaires, hands-on clinic assessments, biological samples, linkage to routine information, abstraction from medical records and environmental monitoring. At baseline the ALSPAC cohort was broadly representative of the UK population, although ethnic minority groups are slightly under-represented [47]. Ethical approval for the study was obtained from the ALSPAC Law and Ethics Committee and the Local Research Ethics Committees.

The pet ownership data has not been previously analysed and are described here in detail. The carer of the child (usually the mother) was asked ‘do you have any pets’ and ‘how many of the following pets do you have’. Pet types included cats, dogs, rabbits, rodents (mice, hamster, gerbil etc), birds (budgerigar, parrot etc) and ‘other’ pets. However, from 2 years onwards a further two categories were added; fish, and turtles/tortoises/terrapins. Pet questions were asked during gestation, and at approximate child age 8 months, 21, 33, 47, 85, 97 and 122 months (up to 10 years old). For simplicity, these will be referred to as gestation, 8 months, 2 years, 3, 4, 7, 8 and 10 years. It was assumed that the child also lived with any pets reported by the mother; in 1998/1999 22% of families in Great Britain were lone mother and only 2% lone father [48]. Thus we imply exposure to pets reported as owned by the mother, as comparable to family pet ownership.

### 2.2. Data Analysis

The pet ownership data required considerable cleaning and recoding of missing data types, particularly for the latter years. If no answers were given to a pet ownership question (all pet questions left blank) this was coded as missing data. If boxes were completed for some pet types but not others, the remainder were assumed as “no” and coded to zero. If pet types were indicated but the initial question on whether they had any pets was blank, it was re-coded as having pets. Numbers of each pet type owned were then used to derive binary variables for owning each pet type or not, at each time point.

Advantage was taken of this longitudinal dataset to examine if there are types of children in relation to when their mother reported living with pets. It was hypothesised that there would be at least two types of individuals depending on their history of pet ownership as reported by their mother; never and always. For those individuals with ownership data for all years, two-step cluster analysis in SPSS was used to identify groupings of similar individuals within the dataset. The Two-Step cluster method is a scalable cluster analysis algorithm designed to handle very large data sets [49]. Each child was assigned to the best cluster that the function felt the individual belonged in, using the binary outcome yes/no for owning a pet type at each time point. This resulted in the formation of a new ordinal variable describing the individual’s pet ownership pattern over time. The process was repeated for history of dog ownership due to the interest in physical health benefits of dog ownership. A number of potential risk factors and confounding variables (including ownership of other pets) were examined for association with ownership of each pet type at each time point, using chi-squared tests and binary logistic regression (for categorical predictor variables) or T-tests (for continuous predictor variables). For ordinal outcomes, chi-squared and one-way ANOVA were used for categorical and continuous predictor variables respectively. Descriptions of these predictor variables are shown in Table 1.

The variables were then entered into multivariable binary logistic regression models (in SPSS), for outcome of mother reported ownership of each pet type at child age 7 years. Multivariable modelling of factors associated with ownership of ‘any’ pet or ‘other’ pet were not performed, as we felt that it was more appropriate to present results individually for each specific pet type. A model was not built for tortoises/turtles due to the small numbers of these pets. Step-wise backwards elimination, using the likelihood ratio, was used to remove variables from each model. Variables remained in the model if there was good evidence for an association (P < 0.05) or if removal resulted in substantial change to the effect of other variables (10% or greater). Two-way interaction terms between the variable ‘mother owned pets as a child’ and other predictor variables were included if required. It is reasonable to suggest that a mother’s pet ownership history may influence how other factors affect her current pet ownership. The fit of the model was assessed using the Hosmer-Lemeshow statistic. The decision was made not to include ownership of the relevant pet type at nearly 4 years (47 months) in the model building process. We hypothesised that it would be very strongly associated with ownership of that pet at 7 years, especially for longer lived animals. However, due to the fact that other variables, such as socioeconomic measures, are also predictors of pet ownership at 4 years, inclusion of pet ownership at 4 years may attenuate their position or exclude them from the model. Consequently, factors relevant to pet ownership would go undetected.

## 3. Results and Discussion

A consistent pet ownership dataset is now available for this birth cohort. ALSPAC is a well-characterised resource that contains a number of physical and mental health outcomes that can be used by researchers concerned with HAI. The access to possible confounding variables and longitudinal nature of the data will allow future researchers to investigate the possible mechanisms underlying potential associations between pets and well-being.

### 3.1. Pet Ownership Trends during Childhood

**Figure 1.**
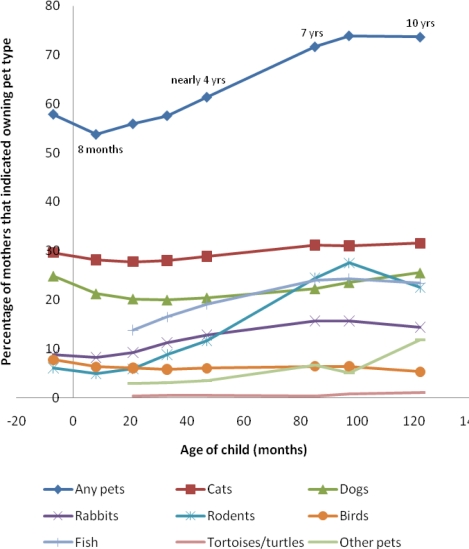
Pet ownership reported by the mother in the ALSPAC cohort. (There are no data on categories ‘fish’ or ‘tortoises/turtles’ for the first two data points and so these and ‘other pets’ category have been excluded from the analysis). Total chi-squared P < 0.001 for all pet types, chi-squared for linear trend P<0.001 for all except dogs (P = 0.05).

Pet information was reported for the mothers of 13,557 children during gestation, and 7,800 by age ten years. Ownership of any pet during gestation was 58%. Family pet ownership of all types changed during different points in childhood (Figure 1). There was an increase over time in ownership of fish, rodents and rabbits. Dog ownership did not follow a linear trend, being 25% at gestation and then dipping in the first few years of childhood. By age 10 years, pet ownership had risen to 74% and dog ownership was 26%. Cats were the most commonly reported pet owned throughout.

Using two-step cluster analysis and including data up to age 7 years, three pet ownership type clusters were identified, subsequently termed: never owned a pet (although some of these owned a pet at age 7 years only); sometimes owned a pet; and always owned a pet (Table 2). Dog ownership also formed three clusters: never owned a dog; sometimes owned a dog (particularly at age 7 years); and always owned a dog (Table 2). When considering data from all available years (up to age 10 years), only two clusters each were identified: sometimes owned a pet, and always owned a pet; never owned a dog and sometime owned a dog (Table 2).

### 3.2. Characteristics of Sample at 7 Years Old

The characteristics of the 8,331 study children with reported pet ownership status of the mother at child age7 years (85 months) are described in Table 3.

### 3.3. Univariable Analysis

Ownership of each pet type reported by the mother across all time points up to child age 10 years, were tested for association with the variables of interest. Univariable results are not presented in full due to the later use of multivariable modelling which accounts better for confounding effects. However, we have described univariable findings in short here, because it is important to indicate factors which may appear to be associated with pet ownership, on first glance. Although some findings for ownership of ‘any pets’ are presented here, for interest, we feel that it is more appropriate to focus on factors associated with individual pet types, as these can differ.

#### 3.3.1. Association between Pet Types

On univariable analysis, ownership of some pet types was often shown to be associated with an increased likelihood of ownership of other pet types (data not shown). This is to be expected, as there are likely to be some people who like pets and own a number of different types. For cats, a negative relationship with dog or bird ownership was seen at gestation (data not shown).

#### 3.3.2. Gender of Child

For all pet types except dog and tortoise/turtle, a positive association was seen for ownership by the mother and the child being female compared to male (data not shown). The effect appeared as the children got older.

#### 3.3.3. Ethnicity

Pet ownership reported by the mother differed by ethnicity of the child at all time points: at 7 years, 72% of category ‘white’ owned pets, compared to 59% ‘mixed’, 33% ‘asian’, 15% ‘black’ and 38% ‘other’ (P < 0.001). When categories were combined into ‘white’ and ‘other’, children that were ‘white’ were more likely to own any pets (OR = 2.15, 95%CI 1.63–2.83, P < 0.001). When analysed by separate pet types, the same relationship was seen for most years, except for birds and tortoise/turtle which did not appear to be associated with ethnicity (data not shown).

#### 3.3.4. Number of People in the Household

Larger households (5 or more people) were more likely to own all pet types except cats, and this relationship persisted throughout childhood (data not shown).

#### 3.3.5. Presence of an Older Sibling

Presence of an older sibling was assessed at 18 months of age and assumed to remain constant. It appeared to be consistently positively associated with the mother reporting ownership of all pet types except cats, for which there was a negative association at gestation which then disappeared by 33 months (data not shown). When stratified by whether the child had older siblings or not, the pet ownership trends over time remained similar to that reported above (Figure 1), but generally lower rates of pet ownership were observed for those without an older sibling. However, there was now no evidence for a linear trend (Chi-squared test for trend) for birds by those with an older sibling. For both those with, and without older siblings, there was now stronger evidence of a linear trend for dog ownership than there was before stratification (P < 0.001 compared to P = 0.05).

#### 3.3.6. Maternal and Paternal Education

The general pattern seen over most years was for those who had achieved higher education levels to be less likely to own each pet type than the lowest education level ((CSE)Certificate of Secondary Education/None), however, there was a positive association between higher education level and cat ownership (data not shown).

#### 3.3.7. Maternal and Paternal Social Class

In nearly all years and for most pet types, professional occupations were least likely to own pets and an increasing gradient across decreasing social class could be seen (data not shown). However, for maternal social class, skilled manual were the least likely to own cats.

#### 3.3.8. Maternal Age at Delivery

The mean age at delivery of mothers of children in each pet owning history category (never, sometimes, always—up to 7 years) was compared using one-way analysis of variance. Children whose mothers ‘sometimes had a pet’ had the youngest mothers (P < 0.001). Individual differences were also seen between ownership of different pet types at different years and maternal age (t-tests, data not shown), although direction of the association varied. Maternal age was categorised into <21, 21–30, >30 yrs; mothers <21 yrs being the greatest proportion of ‘sometimes had a pet’ (P < 0.001). Older mothers had the highest proportion of ‘never had dogs’, however the youngest mothers also had the least tendency to have ‘always had dogs’ (P < 0.001).

#### 3.3.9. House Type

There were differences in mother reported pet ownership by house type for ownership of most pet types (data not shown). The common trend was that children living in terraced houses, flats or rooms were less likely to have mothers that own pets than those living in detached houses.

#### 3.3.10. Mother Had Pets as a Child

When the child was 33 months old (nearly 3 years) the carer (usually the mother) was asked, ‘during childhood (up to the age of 16years), did you have any household pets?’ Many (46%) responded that “yes they always had pets”, 44% responded “yes, part of the time”, and only 10% responded “no, not at all”. This was compared with pet ownership reported by the mothers of the children of ALSPAC at different ages (data not shown). Mothers who sometimes owned a pet as a child were more likely to now report owning them than those who never had pets, and the likelihood was even higher for those who had always had pets as a child. This was seen for every year data point and was unaffected by adjustment for maternal education and social class (data not shown). Using the groupings produced by cluster analysis for pet ownership up to 7 years (never, sometimes, always), there was a pattern for those who had pets as a child (sometimes, always) increasingly also having pets when they had children (P < 0.001).

#### 3.3.11. Previous Pet Ownership by Child

On univariable analysis, pet ownership at nearly 4 years (47 months) was a strong predictor of pet ownership at 7 years (85 months) (full data not shown; for cat and dog the likelihoods were increased by 42 times and 49 times respectively). This is partly explained by the fact that dogs and cats are likely to still be alive 3 years later.

### 3.4. Multivariable Models for Age 7 Data

Presenting results in a univariable manner is subject to the effects of confounding factors, which may be on the causal pathway and associated with the independent variable of interest. Multivariable modelling of pet ownership data better accounts for confounding factors, thus previously apparent associations can disappear.

#### 3.4.1. Cat Ownership

The final multivariable model of cat ownership at 7 years is presented in Table 4, alongside univariable results for comparison. Children were more likely to have a mother reporting to own a cat if they: also owned another pet of the ‘other pet’ category; were female or were white. There was an interaction term required between maternal education and mother owning pets as a child: if the mother sometimes or always had a pet as a child, those with only vocational or O-level (equivalent to GCSE) qualifications were the most likely to own a cat; but if the mother had never had pets as a child, those with only vocational or O-level qualifications were least likely to own a cat. The Hosmer-Lemeshow statistic was very high, suggesting good model fit.

#### 3.4.2. Dog Ownership

The final multivariable model of dog ownership at 7 years is presented in Table 5, alongside univariable results for comparison. Children were more likely to have a mother reporting to own a dog if they also owned a bird, fish, or ‘other’ pet. The likelihood of dog ownership increased with 5 or more people in the household. Children with an older sibling (at 18 months) were more likely to have a mother that reported owning a dog, and this association is independent of number of people in the household, which might be expected to partly explain each other. The younger the mother was at delivery, the more likely she was to be living with a dog. There were also differences in dog ownership between paternal social class types, with part-skilled occupations more likely to own a dog than professional occupations. For maternal social class, unskilled occupations were more likely to own a dog than professionals. Living in a semi-detached, end terraced, terrace, or flat/other reduced the odds of owning a dog compared to living in a detached house. There was an interaction between mother owning pets as a child and paternal education: where the mother had never owned pets, as paternal education increased the likelihood of dog ownership decreased. However, the same was not true when the mother had owned pets as a child.

#### 3.4.3. Rabbit Ownership

The final multivariable model of rabbit ownership at 7 years is presented in Table 6, alongside univariable results for comparison. Children were more likely to have a mother that reported owning a rabbit if they also owned a rodent, bird, fish, other pet, were female, had 5 or more people in the household, or their mother had owned pets as a child. Those with maternal education at degree level, maternal age over 30 years, or that lived in a flat/room/other, were less likely to own rabbits than those with the lowest education, maternal age less than 21 years, or living in a detached house, respectively.

#### 3.4.4. Rodent Ownership

The final multivariable model of rodent ownership at 7 years is presented in Table 7, alongside univariable results for comparison. Children were more likely to have a mother that reported owning a rodent if they: owned a bird, owned a fish, were female, were white, had older siblings, had paternal education to O’level or degree level (compared to CSE/none), or their mother owned pets as a child.

#### 3.4.5. Bird Ownership

The final multivariable model of bird ownership at 7 years is presented in Table 8, alongside univariable results for comparison. Children were more likely to have a mother that reported owning a bird if they: had a dog, rabbit, rodent, fish, other pet or older siblings. Likelihood of owning a bird decreased with increasing maternal education level, and was highest in skilled manual, part-skilled and unskilled occupations. Bird ownership was the only pet type that does not appear to be affected by whether the mother owned pets as a child.

#### 3.4.6. Fish Ownership

Unfortunately the Hosmer-lemeshow statistic for the model of fish ownership at 7 years, was very low (0.006), suggesting that the model was not a good fit. Therefore it has not been presented.

### 3.5. Discussion

The ALSPAC sample is of mothers recruited in pregnancy and follows their children from 0–10 years only. It is a sample of one population of children in the UK, and thus may not be generalisable to other areas. Participants are predominately white, but at baseline the ALSPAC cohort was broadly representative of the UK population [47]. Interpretation of findings must note the difference between this population and other UK pet ownership studies which mainly report findings from community or general population-based samples of adults [29,31,33]. The dataset has some advantages over previous ones because of its large sample size, longitudinal design, and use of multivariable analysis including a variety of different factors. The resulting data are powerful, and in part responsible for the discovery of new pet ownership risk factors and interaction terms. It also means that variables with relatively small effect sizes have been identified, which may lead to questioning whether such small effects are substantially useful in predicting pet ownership. Some models were simpler to build and some had better model fit than others. Interaction terms suggested here require cautious interpretation and there is a need to investigate whether these models, and their interaction terms, replicate in other datasets. Interpretation of our data leads to the conclusion that factors contributing to ownership of different pet types are complex and despite our inclusion of factors hypothesised or previously indicated to be associated with pet ownership, there are probably other important factors that were unmeasured and unaccounted for in our analysis..

Models were built without the use of a variable to indicate ownership of the same pet type at the earlier time point, 47 months. Final models were tested with the addition of this variable (data not shown). As hypothesised it was a strong predictor but had the effect of removing some other important variables from the model; mostly factors such as ethnicity, maternal age at delivery, and socioeconomic indicators (education or social class). Although the decision was made to exclude this variable from the model for the reasons above, it may have resulted in over-adjustment due to its place on the causal pathway.

Family pet ownership was seen to increase during childhood; this may be in part due to the prompting effect of asking about more types of pets from 21 months onwards, however this seems unlikely due to the increases mainly being in rodents and rabbits, which were asked in all years. Dog ownership did not follow a linear trend and appeared to dip in the first few years of childhood, which concurs with findings of other studies in the UK and Ireland, namely dog ownership in general is associated with having children in the house, but this is due to the effect of older children (school age), whereas those with young children are less likely to own a dog [29,30,31]. Stratification by presence of older siblings suggested that the association between dog ownership and age became stronger (and now linear), within those with, or without, siblings. This supports the suggestion that dog ownership increases with age of the child.

Pet ownership by the mothers during gestation was fairly high already considering that data were collected during 1990/1991; for comparison, pet ownership in general in the UK has increased over the years and by 2004/2005 it was estimated that approximately half of all UK households owned a pet and a quarter owned a dog [29,50]. Cat ownership was the most commonly reported pet owned throughout, which again was unusual as dogs are often thought to be the most common pet type owned by households in the UK [29,31,51].

Previous studies in the UK and Ireland suggested associations between cat ownership and dog ownership, although their results are conflicting [30,31,33]; we found no association with dog ownership in our final model of cat ownership, and vice versa. An association between dog ownership and fish is interesting as it has been found in a previous study where it disappeared on multivariable analysis [29]. Westgarth [29] also reported an association between dog ownership and horse ownership; horses, if considered a ‘pet’ by respondents, are likely to have been included in our ‘other pets’ category. Birds were found to be associated with multiple compared to single dog ownership in Westgarth *et al.*’s 2007 study [29], but not dog ownership in general, so we suggest that this finding may be related to the multiple dog households in our study.

The relationship between cat or dog ownership and education level of the owners might be more complex than previously reported. Murray [31] reported that cat owners had higher education levels than those without cats, but Eller [34] reported the opposite association; however neither considered whether the respondents had owned pets as a child, with which we found an interaction term. Both Murray [31] and Eller [34] reported that dog ownership decreased as education level increased, however, again they did not report the inclusion of interaction terms. We found an interaction between the mother owning pets as a child and paternal education; where the mother had never owned pets, as paternal education increased the likelihood of dog ownership decreased (similar to that reported in [31,34]), but we found that this relationship was not seen when the mother had owned pets as a child. Associations between dog ownership with social class were reported in Downes *et al.*’s 2009 study [30]. No previous studies have modelled both education and social class (occupation) as predictors of pet ownership, they usually choose one or the other, but our model of dog ownership suggests that they might have independent effects.

After multivariable modelling, maternal age at delivery was only independently associated with dog or rabbit ownership, with likelihood decreasing as maternal age increased. It is unlikely to be due to socioeconomic differences between mothers who give birth when they are older or younger, as these were also included in the model. When analysed using the categories never, sometimes, or always owned a pet (up to age 7 years) it appeared that the youngest mothers were more likely to ‘sometimes’ own a pet, however this detail could not be included in the final models due to the ordinal outcome used.

Previous research suggests that children with younger siblings have fewer pets than those with no younger siblings or singletons [52]. It has also been suggested that the youngest sibling plays more with a pet [53]. In our study, presence of an older sibling appeared to be an independent predictor of dog, rodent, bird and fish ownership by the mother. We defined pet ownership as any pet in the household reported by the mother, whereas previous findings were concerned with pets ‘owned’ by individual children, of which we did not have information. Our results are likely due to the effect of presence of older siblings having an additive effect on the likelihood of pets being in the house, as pet ownership increases as children get older. It may also be explained if having an older sibling increases your likelihood of being a youngest child (with no younger siblings) or, conversely, having no older siblings makes it more likely (over time) that you will acquire at least one younger sibling. In contrast to our findings, a meta-analytical study of birth cohorts on asthma and allergy reported that elder siblings reduced the odds to own cats, but not dogs [34].

Although gender appeared to be associated with a number of pet types on univariable analysis, it did not always remain in the model as it did for cat, rabbit and rodent ownership only (females more likely). Girls may influence their parents to own certain types of pets. The finding that females were more likely to own cats than males concurs with other studies in the UK and Ireland [30,31,33], however this relationship has never previously been demonstrated to also apply to children.

Downes (2009) reported differences in cat ownership by house type, but this did not remain in our model. House type appeared to affect only dog and rabbit ownership in our study. This could be explained by reasoning that dogs and rabbits are perceived to require more outdoor, and possibly indoor, space than other pet types. Number of people in the household was also reported to be associated with dog ownership in the UK in [29] and [31], but we did not see a similar association for cat ownership, again concurring with previous findings [31,33]. Ethnicity other than ‘white’ appears to be associated with decreased cat, or rodent, ownership, as it remained in the models, contrary to our models for some other pet types. This may be due to the power to detect differences due to lower numbers of some other pet types, or real differences in pet keeping by different cultures. It must be noted that in ALSPAC, prevalence of ethnic minorities is relatively low [47].

A general theme was that pet ownership by the mother as a child was a predictor in all but one model (bird); usually owning pets as a child increased the likelihood of pet ownership later on. Although past pet ownership is known to be linked to future pet ownership [54,55], this variable has not always included in studies of factors associated with pet ownership. A limitation of our data set was that we do not know the individual pet types that were owned previously. Pet ownership in one generation appears, in part, to influence ownership in the next generation; that is, people who grow up with pets allow their children to grow up with pets. One explanation is that the characteristics that make some people like pets may be heritable. Alternatively, the will or desire to own a certain pet may overcome what might be perceived as barriers to ownership for other people. For example, if you really like and want a dog, you own one, regardless of lifestyle factors. A significant contributor to this may be because you had dogs as a child.

When describing pet ownership in a sample, a case definition of what constitutes ‘owning’ a ‘pet’ is also important. This interpretation is likely to vary between geographic locations and cultures. Some studies are not as clear as others as to which criteria were used to define ownership of a pet (or even describe how the question was worded in their questionnaire). A random telephone survey in Ireland defined a pet dog as a dog that was being fed by the household and considered a pet by the eligible participant, and a pet cat as a cat that was both fed by the household and allowed into the house [30]. Some differences across study findings may be due to differences in exclusion and inclusion criteria to define a pet, which may or may not include stray, free-roaming, or part-owned animals. In our study, interpretation of what defined whether an animal was a ‘pet’ lay with the respondent (the mother), but they were prompted by a list of common animals considered as pets. The word ‘own’ was not used in the question, which could affect the interpretation. Somebody may live with a pet that is considered ‘owned’ by a different member of the household, so use of this word could confuse interpretation by some respondents. We have also not considered more complex family arrangements where a child shares time between two homes. Although we had to make the assumption that children in this study live with the pets that the mother reported, it would be interesting to investigate whether a child feels that they also own each pet reported by the mother. Due to our assumptions we may have over-estimated or under-estimated the effects of some variables as it is possible that some children, although likely a minority, do not actually live in the same household as the mother, thus do not really live with these pets.

Many people grow up with pets, thus for even a small effect size, the population attributable risk of any health benefit may be of significant value [28]. Three possible explanations are proposed to explain the apparent positive effects of pets on human health [3]: (a) there is no real association and it is due to co-factors that are linked to both pet ownership and health measures; (b) effects are indirect due to enhancement of social contact with people due to proximity to the animal; (c) effects are due to the nature of the relationship with the animal and the provision of emotional support. It is probable that any real effects are mediated through a combination of both (b) and (c), however, due to the quality of study design and data analysis used in some papers championing the positive health benefits of pets, it is difficult to argue that (a) is not the case.

This study demonstrates clearly that there are a number of socioeconomic and demographic factors that are associated with pet ownership, thus the relevant ones to specific pet types must also be accounted for in data analysis of any possible improved health outcomes from owning pets. Socioeconomic factors are known to be associated with the health of individuals, for example, in children of the ALSPAC sample, both fat mass [56] and number of doctor’s consultations [57] differed by socioeconomic indicators. In our analyses, education and social class often had independent effects on pet ownership, and should therefore both be included if possible, rather than one as a proxy for both.

It is clear that the factors contributing to the ownership of different pet types can be very different, and so it is very important to consider these separately rather than lumping as ‘pet ownership’. This is particularly relevant when investigating the possible ‘effects’ of pet ownership as a whole, which may be misleading. It has been common practice in HAI research to report the benefits of ‘pet ownership’, when only dogs were studied, or the type of pet studied was not even reported [7]. Considering our findings, this is of concern.

## 4. Conclusions

Pet ownership is a common characteristic of household environments that may affect health. Factors associated with pet ownership differ by pet type and thus they should not be assumed to be the same. These factors are potential confounders in public health research and must be considered when studying the health benefits of pets. Ownership of a ‘pet’ is an artificial construct summarising ownership of a variety of different animals and researchers are advised to consider pet types separately; the human-animal relationship is likely to differ. Children are a significant group of pet owners that are yet to receive much attention; ALSPAC is a resource for research into the effects of pet ownership in children. Researchers designing new cohort studies should seriously consider including questions about pet ownership.

## Figures and Tables

**Table 1.** Potential risk factors and confounders, method and time of data collection, and factor level of analysis.

**Variable**	**Method and time of data collection**	**Level**
Cat	Derived from number of cats in mother’s questionnaire at gestation, 8, 21, 33, 47, 85, 97 and 122 months	No, yes
Dog	Derived from number of dogs in mother’s questionnaire at gestation, 8, 21, 33, 47, 85, 97 and 122 months	No, yes
Rabbit	Derived from number of rabbits in mother’s questionnaire at gestation, 8, 21, 33, 47, 85, 97 and 122 months	No, yes
Rodent	Derived from number of rodents in mother’s questionnaire at gestation, 8, 21, 33, 47, 85, 97 and 122 months	No, yes
Bird	Derived from number of birds in mother’s questionnaire at gestation, 8, 21, 33, 47, 85, 97 and 122 months	No, yes
Fish	Derived from number of fish in mother’s questionnaire at gestation, 8, 21, 33, 47, 85, 97 and 122 months	No, yes
Tortoises/turtles	Derived from number of tortoises/turtles in mother’s questionnaire at gestation, 8, 21, 33, 47, 85, 97 and 122 months	No, yes
Other pet	Derived from number of other pets in mother’s questionnaire at gestation, 8, 21, 33, 47, 85, 97 and 122 months	No, yes
Gender	Medical records	Male or female
Ethnicity	Carer questionnaire (child-based) at140 months	White, mixed, asian, black, other. Collapsed to ‘white’ and ‘other’.
Number of people in household	Derived from mother’s questionnaire at gestation, 8, 21, 33, 47,85, 97, 122 months.	3, 4, 5+
Presence of an older sibling	Derived from mother’s questionnaire (child based) at 18 months	No, yes
Maternal education	Mother’s questionnaire at 32 weeks gestation. Highest level indicated.	CSE or no qualification (lowest), vocational, O level, A level, degree (highest)
Paternal education	Mother’s questionnaire at 32 weeks gestation. Highest level indicated.	CSE or no qualification (lowest), vocational, O level, A level, degree (highest)
Maternal social class	Derived from mother’s questionnaire at 32 weeks gestation (occupation).	Professional (highest), Managerial and technical, Skilled:non-manual, Skilled:manual, Partly skilled, Unskilled (lowest).
Paternal social class	Derived from mother’s questionnaire at 32 weeks gestation (occupation).	Professional (highest), Managerial and technical, Skilled:non-manual, Skilled:manual, Partly skilled, Unskilled (lowest).
Maternal age at delivery	Clinical records	Continuous (years) OR < 21 years, 21–30 years, >30 years.
Mother had pets as a child	Mother’s questionnaire at 33 months.	No, not at all; Yes, part of time; Yes, always.
House type	Derived from mother’s questionnaire at gestation, 8, 21, 33,85, 122 months	Detached, semi-detached, end terrace, terraced, flat/room in someone else’s house/other (*note for gestation no end terrace category*)

**Table 2.** Two-step cluster analysis in SPSS to identify mother reported pet-ownership types (from 14,663 fetuses).

**Variable**	**Included data (%)**	**Cluster name**	**Distribution (%)**
Any pet—up to (and including) 7yrs	46	Never owned a pet	25
		
		Sometime owned a pet	38
		
		Always owned a pet	37

Dog—up to (and including) 7yrs	46	Never owned a dog	68
		
		Sometime owned a dog	17
		
		Always owned a dog	15

Any pet—all years (up to and including 10yrs)	37	Sometimes owned a pet	53
		
		Always owned a pet	47

Dog—all years (up to and including 10yrs)	37	Never owned a dog	64
		
		Sometime owned a dog	36

**Table 3.** Characteristics (number and percentage) of the study children at 7 years (85 months).

Variable (*=specific to 85 months)	**Level**	**Number**	**Percentage**
Any pet	Yes	5,972	72%
Cat	Yes	2,594	31%
Dog	Yes	1,855	22%
Rabbit	Yes	1,305	16%
Rodent	Yes	2,037	24%
Bird	Yes	534	6%
Fish	Yes	1,993	24%
Tortoises/turtles	Yes	26	0%
Other pet	Yes	557	7%
Gender*	Male	4,312	52%
	Female	4,019	48%
Ethnicity*	White	6,068	97%
	Mixed	175	3%
	Asian	15	0.2%
	Black	13	0.2%
	Other	8	0.1%
	Other than white combined	211	3%
Number of people in household*	3	1,233	15%
	4	4,168	50%
	5+	2,904	35%
Presence of an older sibling at 18 months*	No	3,636	46%
	Yes	4,323	54%
Maternal education*	CSE or no qualification (lowest)	1,145	14%
	Vocational	710	9%
	O level	2,873	35%
	A level	2,102	26%
	Degree (highest)	1,269	16%
Paternal education*	CSE or no qualification (lowest)	1,631	21%
	Vocational	639	8%
	O level	1,711	22%
	A level	2,199	28%
	Degree (highest)	1,683	21%
Maternal social class*	Professional (highest), Managerial and technical	478	7%
		2,365	34%
	Skilled: non-manual	2,957	43%
	Skilled: manual	467	7%
	Partly skilled	550	8%
	Unskilled (lowest)	116	2%
Paternal social class*	Professional (highest), Managerial and technical	941	13%
		2,667	36%
	Skilled: non-manual	858	12%
	Skilled: manual	2,154	29%
	Partly skilled	603	8%
	Unskilled (lowest)	189	3%
Maternal age at delivery*	<21 years	303	4%
	21–30 years	5,043	61%
	>30 years	2,985	36%
Mother had pets as a child*	No, not at all	743	10%
	Yes, part of time	3,517	46%
	Yes, always	3,365	44%
House type*	Detached	2,443	29%
	Semi-detached	3,086	37%
	End terrace	771	9%
	Terraced	1,652	20%
	Flat/room in someone else’s house/other	336	4%

* = Figures given for children with a reported status that their mother owned ‘any pets’ at 85 months (n = 8331) therefore eligible for multivariable modeling of pet ownership outcome; numbers may vary slightly for each pet type due to missing data for that pet type.

**Table 4.** Multivariable binary logistic regression model of cat ownership at 7 years.

**Variable**		**Univariable result (unadjusted)**			**Final adjusted model**	
	**OR**	**95%CI**	**P**	**OR**	**95%CI**	**P**
**Other pet**						
No	1			1		
Yes	1.62	1.36–1.93	<0.001	1.43	1.16–1.78	<0.001
**Gender**						
Male	1			1		
Female	1.09	0.99–1.19	0.08	1.14	1.02–1.28	0.03
**Ethnicity**						
White	1			1		
Other	1.69	1.21–2.36	0.002	0.62	0.43–0.89	0.01
**Interaction—Mother pets as a child × maternal education**						0.04
*Mother pets as a child*						
*No, not at all*	*1*		*<0.001*			
CSE/None	1		0.003	1		
Vocational	1.06	0.87–1.30	0.56	0.42	0.14–1.22	0.11
O Level	1.02	0.88–1.19	0.80	0.33	0.16–0.70	0.004
A Level	1.27	1.09–1.49	0.003	1.05	0.54–2.04	0.89
Degree	1.06	0.89–1.27	0.50	0.72	0.36–1.46	0.36
*Yes, part of time*	*1.77*	*1.44–2.18*	*<0.001*			
CSE/None				1.13	0.62–2.07	0.69
Vocational				2.65	0.84–8.39	0.10
O Level				3.13	1.40–7.02	0.01
A Level				1.08	0.52–2.26	0.83
Degree				1.50	0.69–3.27	0.30
*Yes, always*	*3.43*	*2.79–4.22*	*<0.001*			
CSE/None				1.86	1.04–3.34	0.04
Vocational				3.09	1.00–9.53	0.05
O Level				3.35	1.52–7.36	0.003
A Level				1.40	0.69–2.87	0.36
Degree				1.99	0.93–4.27	0.08

Hosmer-Lemeshow statistic = 0.99, n = 5,818.

**Table 5.** Multivariable binary logistic regression model of dog ownership at 7 years.

**Variable**		**Univariable result (unadjusted)**			**Final adjusted model**	
	**OR**	**95%CI**	**P**	**OR**	**95%CI**	**P**
**Bird**						
No	1			1		
Yes	2.78	2.32–3.32	<0.001	2.17	1.70–2.76	<0.001
**Fish**						
No	1			1		
Yes	1.55	1.38–1.74	<0.001	1.36	1.17–1.57	<0.001
**Other pet**						
No	1			1		
Yes	3.15	2.64–3.75	<0.001	2.24	1.78–2.81	<0.001
**Number of people in household**						
3	1			1		0.03
4	1.07	0.91–1.26	0.40	1.08	0.87–1.34	0.47
5+	1.50	1.27–1.77	<0.001	1.30	1.03–1.64	0.03
**Older sibling at 18 months**						
No	1			1		
Yes	1.47	1.32–1.64	<0.001	1.33	1.14–1.45	<0.001
**Maternal social class**						
Prof	1		<0.001	1		<0.001
Man and tech	1.28	0.98–1.68	0.08	1.00	0.73–1.37	0.98
Skilled NM	1.45	1.11–1.89	0.01	0.91	0.65–1.26	0.56
Skilled M	2.41	1.75–3.32	<0.001	1.36	0.92–2.03	0.12
Part skill	2.44	1.79–3.32	<0.001	1.23	0.84–1.81	0.29
Unskilled	4.12	2.64–6.44	<0.001	2.24	1.29–3.89	0.004
**Paternal social class**						
Prof	1		<0.001	1		0.002
Man and tech	1.17	0.96–1.43	0.11	0.87	0.68–1.12	0.29
Skilled NM	1.36	1.07–1.73	0.01	1.08	0.80–1.46	0.60
Skilled M	1.92	1.57–2.35	<0.001	1.18	0.90–1.55	0.24
Part skill	2.21	1.72–2.83	<0.001	1.43	1.03–2.00	0.04
Unskilled	2.62	1.84–3.71	<0.001	1.38	0.86–2.20	0.18
**Maternal age at delivery**						
<21 yrs	1		<0.001	1		0.02
21–30 yrs	0.84	0.64–1.09	0.18	0.81	0.51–1.27	0.35
>30 yrs	0.65	0.50–0.85	0.002	0.67	0.42–1.06	0.09
**House Type**						
Detached	1		0.07	1		<0.001
Semi–detached	0.99	0.87–1.12	0.88	0.68	0.57–0.80	<0.001
End Terrace	0.98	0.81–1.19	0.86	0.69	0.54–0.89	0.004
Terrace	0.88	0.76–1.03	0.10	0.57	0.47–0.70	<0.001
Flat/room/other	0.68	0.51–0.92	0.01	0.50	0.33–0.77	0.001
**Interaction—Mother pets as a child × paternal education**						0.02
*Mother pets as a child*						
*No, not at all*	*1*		*<0.001*			
CSE/None	1		<0.001	1		
Vocational	0.94	0.77–1.16	0.58	0.80	0.29–2.20	0.67
O Level	0.76	0.65–0.89	0.001	0.73	0.36–1.51	0.40
A Level	0.71	0.61–0.83	<0.001	0.30	0.14–0.63	0.001
Degree	0.40	0.33–0.47	<0.001	0.29	0.13–0.65	0.003
*Yes, part of time*	*1.37*	*1.09–1.74*	*0.01*			
CSE/None				1.04	0.61–1.78	0.88
Vocational				1.06	0.36–3.17	0.91
O Level				0.91	0.41–2.01	0.82
A Level				2.04	0.92–4.52	0.08
Degree				1.25	0.53–2.94	0.61
*Yes, always*	*2.97*	*2.36–3.74*	*<0.001*			
CSE/None				1.46	0.87–2.47	0.15
Vocational				1.32	0.45–3.87	0.61
O Level				1.26	0.58–2.72	0.56
A Level				3.31	1.52–7.20	0.003
Degree				2.41	1.04–5.58	0.04

Hosmer-Lemeshow statistic = 0.32. n = 5,867.

**Table 6.** Multivariable binary logistic regression model of rabbit ownership at 7 years.

**Variable**		**Univariable result (unadjusted)**			**Final adjusted model**	
	**OR**	**95%CI**	**P**	**OR**	**95%CI**	**P**
**Rodent**						
No	1			1		
Yes	1.51	1.33–1.72	<0.001	1.23	1.06–1.42	0.01
**Bird**						
No	1			1		
Yes	1.91	1.56–2.35	<0.001	1.44	1.14–1.82	0.002
**Fish**						
No	1			1		
Yes	1.82	1.60–2.07	<0.001	1.65	1.43–1.90	<0.001
**Other pet**						
No	1			1		
Yes	1.84	1.50–2.25	<0.001	1.56	1.24–1.95	<0.001
**Gender**						
Male	1			1		
Female	1.28	1.13–1.44	<0.001	1.33	1.17–1.51	<0.001
**Number of people in household**						
3	1			1		0.06
4	1.30	1.07–1.58	0.01	1.11	0.90–1.38	0.33
5+	1.65	1.35–2.00	<0.001	1.28	1.02–1.60	0.04
**Maternal education**						
CSE/None	1		<0.001	1		<0.001
Vocational	0.95	0.74–1.21	0.66	1.09	0.83–1.43	0.54
O Level	0.95	0.79–1.14	0.57	1.06	0.86–1.30	0.58
A Level	0.81	0.67–0.99	0.04	0.92	0.74–1.14	0.43
Degree	0.48	0.37–0.60	<0.001	0.62	0.47–0.81	<0.001
**Maternal age at delivery**						
<21 yrs	1		0.04	1		0.03
21–30 yrs	0.97	0.71–1.32	0.85	0.75	0.51–1.10	0.13
>30 yrs	0.83	0.60–1.13	0.24	0.64	0.43–0.95	0.03
**Mother pets as a child**						
No, not at all	1		<0.001	1		<0.001
Yes, part of time	1.55	1.19–2.00	0.001	1.46	1.11–1.91	0.01
Yes, always	2.02	1.56–2.61	<0.001	1.75	1.34–2.28	<0.001
**House type**						
Detached	1		<0.001	1		0.04
Semi–detached	1.13	0.98–1.31	0.09	1.04	0.88–1.22	0.67
End Terrace	1.14	0.92–1.42	0.24	0.98	0.76–1.25	0.86
Terrace	1.03	0.87–1.23	0.72	0.96	0.79–1.16	0.66
Flat/room/other	0.47	0.31–0.71	<0.001	0.50	0.31–0.79	0.003

Hosmer-Lemeshow statistic = 0.16. n = 7,206.

**Table 7.** Multivariable binary logistic regression model of rodent ownership at 7 years.

**Variable**		**Univariable result (unadjusted)**			**Final adjusted model**	
	**OR**	**95%CI**	**P**	**OR**	**95%CI**	**P**
**Bird**						
No	1			1		
Yes	1.86	1.55–2.23	<0.001	2.06	1.63–2.61	<0.001
**Fish**						
No	1			1		
Yes	1.79	1.60–2.00	<0.001	1.70	1.48–1.95	<0.001
**Gender**						
Male	1			1		
Female	1.09	0.99–1.21	0.08	1.19	1.05–1.34	0.01
**Ethnicity**						
White	1			1		
Other	2.32	1.53–3.51	<0.001	0.51	0.32–0.81	0.01
**Older sibling at 18 months**						
No	1			1		
Yes	2.03	1.83–2.26	<0.001	1.97	1.73–2.24	<0.001
**Paternal education**						
CSE/None	1		0.64	1		0.02
Vocational	0.92	0.74–1.15	0.47	0.93	0.70–1.23	0.61
O Level	1.05	0.90–1.23	0.51	1.23	1.01–1.51	0.04
A Level	0.97	0.84–1.13	0.72	1.06	0.88–1.28	0.55
Degree	1.05	0.90–1.23	0.55	1.29	1.06–1.57	0.01
**Mother pets as a child**						
No, not at all	1		<0.001	1		<0.001
Yes, part of time	1.61	1.31–1.99	<0.001	1.62	1.27–2.08	<0.001
Yes, always	1.94	1.31–1.99	<0.001	1.91	1.49–2.45	<0.001

Hosmer-Lemeshow statistic = 0.67, n = 5,611.

**Table 8.** Multivariable binary logistic regression model of bird ownership at 7 years.

**Variable**		**Univariable result (unadjusted)**			**Final adjusted model**	
	**OR**	**95%CI**	**P**	**OR**	**95%CI**	**P**
**Dog**						
No	1			1		
Yes	2.78	2.32–3.32	<0.001	2.18	1.75–2.72	<0.001
**Rabbit**						
No	1			1		
Yes	1.91	1.56–2.35	<0.001	1.56	1.22–2.00	<0.001
**Rodent**						
No	1			1		
Yes	1.86	1.55–2.23	<0.001	1.62	1.30–2.03	<0.001
**Fish**						
No	1			1		
Yes	1.80	1.50–2.17	<0.001	1.46	1.17–1.83	0.001
**Other pet**						
No	1			1		
Yes	2.35	1.80–3.06	<0.001	1.84	1.34–2.53	<0.001
**Older sibling at 18 months**						
No	1			1		
Yes	1.83	1.51–2.22	<0.001	1.34	1.08–1.67	0.01
**Maternal education**						
CSE/None	1		<0.001	1		<0.001
Vocational	0.72	0.53–1.00	0.05	0.92	0.62–1.37	0.68
O Level	0.55	0.43–0.69	<0.001	0.73	0.54–0.99	0.05
A Level	0.37	0.28–0.49	<0.001	0.50	0.35–0.72	<0.001
Degree	0.24	0.16–0.34	<0.001	0.39	0.24–0.65	<0.001
**Maternal social class**						
Prof	1		<0.001	1		0.01
Man and tech	1.21	0.72–2.04	0.47	0.85	0.47–1.53	0.58
Skilled NM	1.67	1.00–2.77	0.05	0.88	0.48–1.62	0.67
Skilled M	2.47	1.38–4.43	0.002	1.22	0.62–2.41	0.57
Part skill	3.63	2.10–6.29	<0.001	1.48	0.77–2.85	0.24
Unskilled	5.31	2.66–10.59	<0.001	1.82	0.81–4.06	0.15

Hosmer-Lemeshow statistic = 0.80. n = 6,655.
